# System based greenhouse emission analysis of off-site prefabrication: a comparative study of residential projects

**DOI:** 10.1038/s41598-023-37782-x

**Published:** 2023-07-01

**Authors:** Yuliang Guo, Enhui Shi, Rui Yan, Wenchao Wei

**Affiliations:** 1 School of Economics and Management, Beijing Jiaotong University, Beijing, China; 2 School of Economics and Management, University of Science and Technology Beijing, Beijing, China

**Keywords:** Environmental sciences, Environmental social sciences, Mathematics and computing

## Abstract

High-story residential structures and off-site prefabrication have been dominant choices in the construction industry. There is a substantial quantity of greenhouse gas (GHG) emissions produced by the construction industry. In fact, the construction industry is responsible for 30 percent of all GHG emissions. In this study, we analyse the differences between the conventional technique of building and the off-site prefabricating construction method. First, we evaluate the emissions emitted from key processes during the off-site prefabricating construction. In addition, we analyse the qualitative and quantitative differences between two prefabrication structural systems, namely concrete and steel, which are the two most common structural systems utilised in residential construction projects in China. We examine and analyse four different case studies in order to exemplify the proposed methodology and offer managerial insights.

## Introduction

In Asian countries with a large population, i.e., China, high-story structure is one of the major construction methods of residential projects. It is essential to develop high-story residential structures as the amount of available land in the central regions of large cities, such as Beijing, continues to shrink while the price of land continues to climb. In 2022, the completed residential areas account for 67.36% of the total completion area in China. While the proportion for plant, service, and office are 12.2%, 7.11%, and 4.77%, respectively (China Statistic Yearbook^1^)^2^.

With the emergence of the concept of sustainable construction and development, the construction industry is keen to limit its greenhouse gas (GHG) emissions, since it is the leading contributor that produces the global GHG emissions^3^. According to Kibert^4^, sustainable construction based on ecological principles and resource-efficient is environmentally friendly. The construction sector consumes 40% of the overall energy and therefore it releases around 30% of the total greenhouse gas emissions each year^5^. Huovila et al.^2^ claimed that GHG emissions are set to more than twice in the next 20 years since inefficiencies in the existing building sectors and rapid urbanization. It is imperative to reduce GHG emissions of residential projects and it is now a focus of research^6^.

Pioneer works about this problem extensively revealed the importance of buildings’ life cycle analysis and intensive research in an individual phase of a life cycle^7^. A significant number of studies has focused on the development of innovative technologies, methodologies and policies to mitigate GHG emissions at the operating level^5,8–10^, rather than in the construction phase. Relatively fewer researches were conducted on GHG emissions and their environmental impact. Guggemos and Horvath^11^ pointed out that GHG emissions account for around 12% of the overall effect of the operating phase. On-site construction and off-site prefabrication are the two major construction methods. For the construction material, Cole^12^ examined timber, steel, and concrete structural systems separately to find out whether structural material alternatives make a significant difference during construction. González and Navarro^13^ suggests that the usage of low environmental impact mater could reduce carbon dioxide (CO_2_) emissions by almost 30%. Yan et al.^14^ calculated the GHG emissions in building constructions by proposing a quantitative method. They also argued that materials’ embedded releases are the major contributors of GHG, therefore the use of recycled materials during the construction stage can decrease GHG emissions. We refer to Orsini and Marrone^15^ for an extensive review of the low-carbon production of building materials.

Off-site prefabricating is considered a great construction method with a large potential of reducing GHG emissions^4^. Recently, Aye et al.^16^ made a comparison of the embedded energy usage and GHG emissions among traditional concrete, precast steel, and precast timber building systems. Their study indicates that a proper selection of materials is preferable to the changes in construction processes to reduce environmental impact. Jaillon and Poon^17^ reviewed the development of prefabricated residential buildings in Hong Kong and suggested that the scope of prefabrication is reflected in the proportion of prefabrication by volume and the types of prefabricated elements used. As mentioned by Mao et al.^6^, a detailed approach to evaluate the GHG releases of prefabrication is relatively less in the literature and deserves a dedicated study.

As mentioned by Mahapatra and Gustavsson^18^, there are three major structural systems for prefabricated residential projects in China, prefabricated concrete structure, prefabricated steel structure, and prefabricated timber structure. Since 2016, prefabricated concrete buildings and steel structure buildings have been vigorously advocated by the Chinese government. Precast concrete components, which have affluent raw materials, could be widely used in industrial and residential buildings and have become the mainstream development of new industrialization of architecture. The prefabricated steel structural system has a wide range of applications as well, however, it has a smaller market share because of the higher cost. The prefabrication construction makes up only 3% of the newly constructed buildings, therein the proportion of steel structure buildings is less than 1%. The low-rise public buildings such as schools, kindergartens, nursing homes, gardens, and landscapes are appropriate for adopting prefabricated wood structure systems. Although this type of construction could significantly improve environmental sustainability, insufficient timber has limited the development of prefabricated timber structure buildings.

As described above, high-story residential projects are one of the major sources of GHG emissions in construction industry, and the selection of different construction methods and structures have significant impact of the amount of GHG emission. Our contribution in this paper is threefold: firstly, we aim to quantify the life-cycle GHG emissions of different building systems and to demonstrate whether prefabrication is a favorable and efficient method in GHG emissions reduction approaches; secondly, we investigate the quantitative differences between two prefabrication structural systems: concrete and steel systems, which are the two important structural systems utilized in residential projects in China; thirdly, four case studies are conducted to demonstrate the proposed approach and provide managerial insights.

The paper is structured as follows: Section “Literature review” focus on the literature about the definition of GHG emissions and off-site prefabrication, and the calculation of GHG emissions. Section “Methodology” introduces the system boundary analysis and process-based evaluation used to calculate the emission of residential projects. A comparative analysis of two groups of residential projects with different structures and different construction methods is conducted in Section “Case study”. In Section “Conclusions”, we summarize the conclusions and future research directions.

## Literature review

### GHG emissions

United Nations Framework Convention on Climate Change (UNFCCC) of Kyoto Protocol defined that GHG include carbon dioxide (CO_2_), methane (CH_4_), nitrous oxide (N_2_O), hydro fluorocarbons (HFCs), perfluorocarbons (PFCs) and sulphury hexafluoride (SF6)^19^. The calculation of GHG emissions is considered to be a good index of the total effect of energy usage on the environment. As being one of the oldest sectors, the construction sector is inefficient, wasteful and labor-intensive. Furthermore, its conventional construction methods are environmentally unfriendly due to the large resource consumption, waste production, and GHG emissions. The carbon emissions attributed to buildings are considered a leading factor in global warming. GHG emissions reduction has got a lot of attention in the construction industry. Hong et al.^20^ investigated data on assembly and miscellaneous buildings and human activities during on-site construction and argued that indirect operations contribute to 97% of total GHG emissions. Sandanayake et al.^21^ further studied the GHG and non-GHG emissions during the construction phase of building foundations.

GHGs come from all life-cycle phases of a building. For the construction stage of buildings, many scholars have various classifications of the GHG emissions sources. Guggemos and Horvath^11^ argues that it should include raw materials acquisition and manufacturing, construction, usage, maintenance, and end-of-life. In the study of Upton et al.^22^, the embodied GHG emissions of residential structures include those related to obtaining raw materials, fabricating construction materials, shipping materials to building sites, and constructing the buildings. Then Yan et al.^14^ summarized six types of GHG emissions in the constructing stages, including construction supplies’ fabricating and transporting, construction devices’ transporting, building equipment’s’ energy usage, workers’ transporting, and building waste’s handling.

Comparative studies of residential project has gradually been a focused research topic. Jonkute et al.^23^ analyzed the carbon dioxide emission of residential buildings in Lithuania, through the energy performance certification. A similar study in Egypt can be found in Marey et al.^24^. Luo et al.^25^ studied the carbon emission for the renovation of old residential areas through a life cycle assessment. Based on BIM and life cycle assessment, Yang et al.^26^ conducted a field study of carbon footprint calculation of a deferential project. Mao et al.^6^ provided a comparative study of GHG emissions of residential projects. A comparative carbon footprint analysis between commercial and residential projects is presented in Nuri Cihat et al.^27^. The life-cycle impact of alternative structural materials of prefabricated and conventional construction is discussed in Tavares et al.^28^.

### Off-site prefabrication

Reducing carbon emissions of buildings’ construction has become an urgent and important issue. Numerous strategies such as deconstructive design, lean construction, waste management, and prefabrication have been used to improve constructing efficiency and environmental performance^14,16,29^. Off-site prefabrication offers great promise for environmental sustainability. National Research Council^30^ defined that prefabrication involves two stages, which are the fabrication or assembly of systems and components at off-site manufacturing factories and the shipment and installation for systems or components at the construction job site. The proper usage of these techniques can reduce construction time, reduce costs, and improve project quality.

Some studies have made a comparison between the life-cycle energy usage and GHG emissions of conventional and prefabricated building processes. The results Jaillon et al.^31^ showed that the usage of the prefabricated construction techniques can take a 52% average wastage reduction comparing with the conventional construction method. Aye et al.^16^ concludes that the method of prefabrication can significantly contribute to the cost savings and time efficiency of buildings. In the construction industry, off-site prefabricating methods can be classified into three groups according to whether building components are entirely or partly manufactured in the factory, namely semi-prefabricated, integrated prefabricated, and volumetric modular buildings^32^.

### GHG emissions calculation

Some researchers have attempted to evaluate the environmental influence of the constructing sector and different tools have been employed to quantify these impacts. Current energy analyzing techniques could be basically divided into four different types, statistical method, process-based analysis, input–output (I–O) method, and hybrid analysis method^33^.

The accuracy and scope of analysis methodologies are different. Statistical analysis is considered as an efficient and effective method to acquire the environmental influence of buildings^14,16^. However, the accuracy and applicability of the statistical analysis rely on the quality of the collected data. Besides, the difficulties of acquiring authorized statistics prevent this data-driven method from being widely studied.

The process-based analysis systematically reviews the environmental impact along the (material) production process and provides a bottom-up evaluation approach. It starts with the final state of production and works backward to calculate the energy input that needs to be determined^33^. Chen et al.^34^ evaluated energy use in all life cycle of buildings in Hong Kong. You et al.^35^ integrated a quantitative model to analyze the GHG emissions of life cycles of city building systems. However, the time and effort required is the main disadvantage of this method. It is also possible that particular pieces of data cannot be obtained, which diminishes its practical application.

Moreover, the input–output analysis tackles the evaluation issues from a larger scale. In this top-down method, any relevant industry sectors are considered as the inputs to the evaluation system and then combined to deliver the conclusion. This method is widely adopted to evaluate the GHG emissions of the building constructing industry^36,37^. Nässén et al.^38^ used input- output analysis to evaluate carbon emissions and energy usage in the Swedish building sector, and then compared the results with 18 previous bottom-up results using a process-life-cycle analysis approach. This method is used to evaluate pollution impact on a macroeconomic scale, therefore limits its usage in calculating GHG emissions from a microscope perspective.

Chen et al.^39^ presented a framework for assessing low carbon buildings using a multi-scale input–output analysis procedure. The strength of the input–output analyzing is that it allows for a thorough and structured analyzing of energy demand for any production systems since it fully encompasses the economic system as determined by regional or national statistics. However, the use of national average data can limit the robustness and applicability of the results^16^.

In order to combine the advantages of the above-mentioned methods and try to incorporate the most important features, a few studies combined these methods to improve the assessment quality. Aye et al.^16^ produced an integrated quantitative model to evaluate embodied energy. Seo and Hwang^40^ applied the input–output analysis to assess CO_2_ usage from the building constructing stage and the process-based approach to calculate the rest phases of the building manufacturing. Alcorn and Baird^41^ conducted a mixed analysis of the case of recycled steel and calculated energy embodied factors for a series of building materials. The results indicate that the hybrid approach comprising a process analysis and a supplemented input–output analysis is faster and more accurate than a standalone method.

The advent of prefabrication in China only occurs in recent years. The economical input-out data from the upstream of each stage along the supply chain of prefabrication, are still not available yet. As suggested by Mao et al.^6^, it would be favorable to conduct a microscope approach to evaluate GHG emissions. In this paper, we design a process-based method to quantify the GHG emissions of prefabrication to overcome the deficiencies of input-out data.

## Methodology

In this section, we propose an unified approach to evaluate the GHG emissions of residential projects of different structural systems. A process-based life cycle assessment, which has been used in evaluating GHG emissions of conventional construction projects^11,14,22^, is adopted to analysis the boundaries of environmental impact of residential projects with different structure systems. Then a mathematical evaluation of GHG emissions along the construction process is proposed in Section “Quantitative models”.

### Evaluation system boundary

Life cycle assessment applies to buildings includes the analysis and evaluation of the environmental impact of building elements over their entire life cycle in terms of material manufacturing, building constructing, usage, and dismantling. One of the priorities to successfully conduct the process-based method is to define and consolidate the evaluation system boundary as well as the sources of GHG emissions. Some researchers have tried to assess the GHG emissions in the constructing phase of buildings with different system boundaries. In Yan et al.^14^, the system boundary is limited to the production and transportation of building materials, and the erection of buildings. Mao et al.^6^ limited the calculation components to three groups, which include the GHG usage of materials, fuel combustion, and resource consumption.

The GHG emissions from operating and dismantling are not considered in this paper, as the energy consumption in these two phases in the two compared cases is similar. Besides, the transportation of construction workers is not accounted as well, because in China they usually live on construction sites. The revised building construction processes considered in this paper are:

- Manufacturing and transporting of constructing materials;
- Energy usage of on-site activities;
- Construction waste disposal.

The GHG emission mainly comes from the energy consumptions in these processes. Therefore, within the limits of the system boundary, we have summarized the five sources of GHG emissions as follows:

- *E1* GHG emissions from construction materials;
- *E2* GHG emissions from the fuel usage of construction materials’ transporting, from the material providers to the project constructing plants or from the material providers to the prefabrication factory;
- *E3* GHG emissions from the fuel usage for prefabricated components transportation;
- *E4* GHG emissions from the energy usage and construction activities on the construction site;
- *E5* GHG emissions from the fuel burning for waste disposal transportation (from the project construction site to the land-fill places).

**Figure 1:**
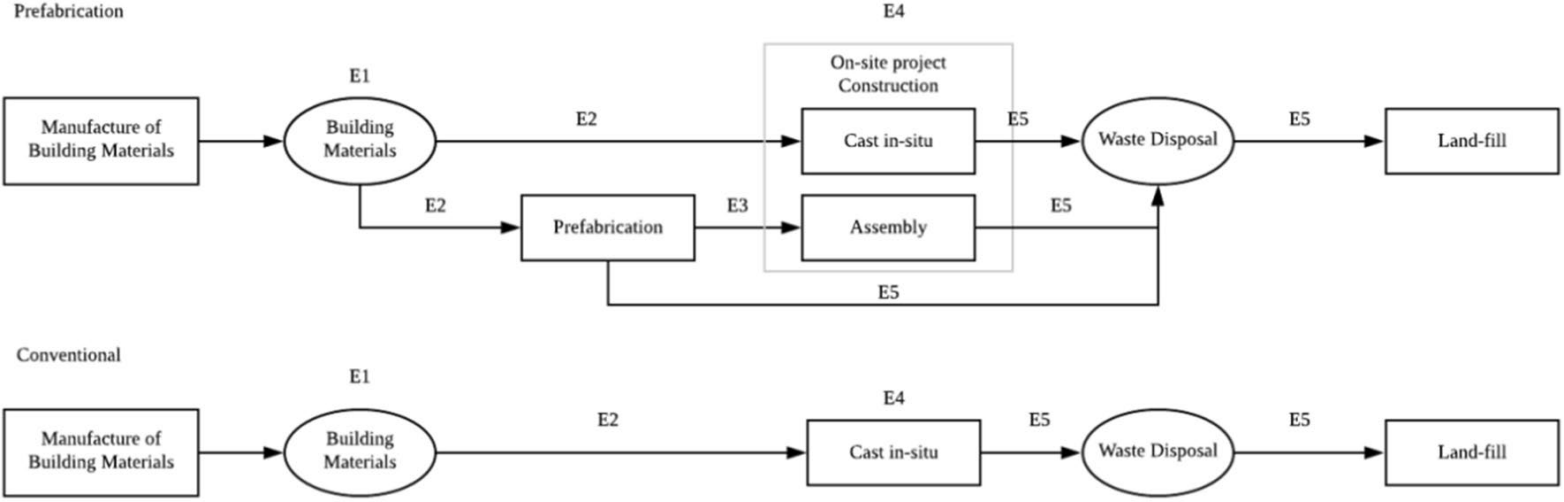
Computational boundaries of GHG emissions in construction stage.

The computational boundaries of the construction process for the prefabricated and conventional methods in this study are showed in Fig. 1. For the prefabrication construction method, some building materials need to be cast in-situ, and some need to be pre-produced in the off-site prefabrication factory. In this case, construction materials need to be transported to the project in-situ and prefabrication factory separately. And then, the additional sources of GHG emissions from prefabricated components’ transporting and waste disposal in the prefabrication factory. By contrast, for conventional construction, all of the building materials are assembled in-situ with a simpler construction process.

Among the six GHG components (as mention in Section “Literature review”), HFCs, PFCs and SF_6_ are seldom emitted in the constructing stages^6^, therefore in this study we focus on the other three components, namely CO_2_, CH_4_ and N_2_O. Besides, each GHG component has different environmental impacts in different dimensions of evaluation^20^.

To overcome this difficulty, most studies use the Global Warming Potential (GWP) value to compare the climatic effect among the various GHG components. Specifically, CO_2_ is used as the reference standard of GHG impacts, and other gases are all converted to CO_2_ equivalents (CO_2−e_). The conversion is based on the GHG emissions factor which is the product of the particular gas emission factor and its GWP value, while the latter one normally has a time span, as the fate of atmospheric changes over time. The current GWP value of CH_4_ and N_2_O are 25 and 298, respectively (fourth assessment report of the IPCC in 2007^3^).

### Quantitative models

According to the above definition of different emission sources, the mathematical models used to calculate GHG emissions during the constructing phase of prefabricated constructions is as follows.

1 $$E_{1} = \mathop \sum \limits_{q} M_{q} *f_{q}^{I}$$

where $E_{1}$ is the total amount of GHG emissions from construction materials (in tons of CO_2−e_), ${M}_{q}$ is the usage of construction material q (in kg), ${f}_{q}^{I}$ is construction material q’s GHG emissions coefficient (in tons CO_2−e_/kg). It should be noted that the value of ${f}_{q}^{I}$ may be interpreted differently in different countries, however, the data of GHG emissions factor is limited in China. In this paper, we collect these factors from the University of Bath’s “Carbon and Energy Inventory”. The third and fourth column of Table 1 present the GHG emissions factors of different construction materials used in the research.

2 $$E_{2} = \mathop \sum \limits_{q} \mathop \sum \limits_{b} \mathop \sum \limits_{k} \frac{{M_{q} *L_{q}^{b} *f_{k}^{\prime \prime } }}{1000}$$

where ${E}_{2}$ is the overall amount of GHG emissions in burning fuel when transporting construction supplies (in tons CO_2−e_), ${L}_{q}^{b}$ is the distance from the material providers to the off-site prefabricating factories for b = 1 or the project construction site for *b* = 2 (in km), and $f_{k}^{\prime \prime }$ is the GHG emissions coefficient (kg CO_2−e_/ton km) for transportation method *k*, with *k* = 1 for truck, 2 for ship, and 3 for train. The GHG emission coefficients for different modes of transport are shown in Table 2.

3 $$E_{3} = \mathop \sum \limits_{j} \mathop \sum \limits_{k} \frac{{P_{j} *L_{j}^{^{\prime}} *f_{k}^{\prime \prime } }}{1000}$$

where ${E}_{3}$ is the overall amount of GHG emissions in the fuel burned in transporting the prefabricated components (tons CO_2−e_), $P_{j}$ is the quantity of prefabricated components (in tons), and $L_{j}^{\prime }$ is the travel distance between the material’s providers and off-site plan (km).

4 $$E_{4} = \mathop \sum \limits_{r} \frac{{R_{r} *f_{r}^{\prime \prime \prime } }}{1000}$$

where $E_{4}$ is the total GHG emissions of fuel consumption and operations on the construction plant (tons CO_2−e_), $R_{r}$ is the overall energy consumption or resources use (in *L*, KWh or $m^{3}$), $f_{r}^{\prime \prime \prime }$ is the GHG emission coefficient of the fuel burning or resources usage (kg CO_2−e_/L, kg CO_2−e_/KW h or kg CO_2−e_/m^3^), where* i* = 1 for the fuel of diesel, 2 for electricity, and 3 for water. Table 3 shows the GHG emission coefficient of energy and resources usage of construction activities.

5 $$E_{5} = \mathop \sum \limits_{q} \mathop \sum \limits_{l} \mathop \sum \limits_{k} \frac{{M_{q} *\delta_{q} *L_{l}^{\prime \prime } *f_{k}^{\prime \prime } }}{1000}$$

where $$E_{5}$$ is the overall GHG emissions in waste disposals’ transporting (tons CO_2−e_), $$\delta_{q}$$ is the factor for waste of the materials *q* generated in the building’s construction (in %). The second column of Table 1 lists the waste factor of different building materials^34,39^. $$L_{l}^{\prime \prime 
}$$ is the travel distance between the construction plant and the off-site plant (in km).

**Table 1:** The GHG emission and waste factors of main building materials.

Building material	Waste factor (%)	CO_2_ emission coefficient (kg CO_2_/kg)	GHG emission coefficient (kg CO_2_−_e_/kg)
Ready-mixed concrete	2.5	0.113	0.120
Cement	2.5	0.653	0.698
Sand	2.5	0.0069	0.007
Steel	5.0	0.352	0.367
Brick	2.5	0.23	0.246
Glass	0	1.735	1.854

**Table 2:** GHG emission factors of different transportation methods.

Mode of transportation	Fuel type	Energy consumption (MJ/ton km)	Fuel CO_2_ emission factor (g CO_2_/MJ)	GHG emission factor (kg CO_2−e_/ton km)
Truck	Diesel	2.423	74.8	0.207
Train	Diesel	0.362	74.8	0.036
Ship	Diesel	0.468	74.8	0.035

**Table 3:** GHG emission coefficients of energy and resources usage.

Type	CO_2_ emission coefficient (kg CO_2_/unit)	GHG emission coefficient (kg CO_2_−e/unit)
Diesel	2.614	2.617
Electricity	0.9489	1.018
Water	–	0.4137

The total GHG emissions during entire constructing stage can be evaluated by Eq. (6), in which is the *i*-th GHG emissions source where *i* is from 1 to 5.

6 $$TGE = \mathop \sum \limits_{i = 1}^{5} E_{i}$$

## Case study

**Figure 2:**
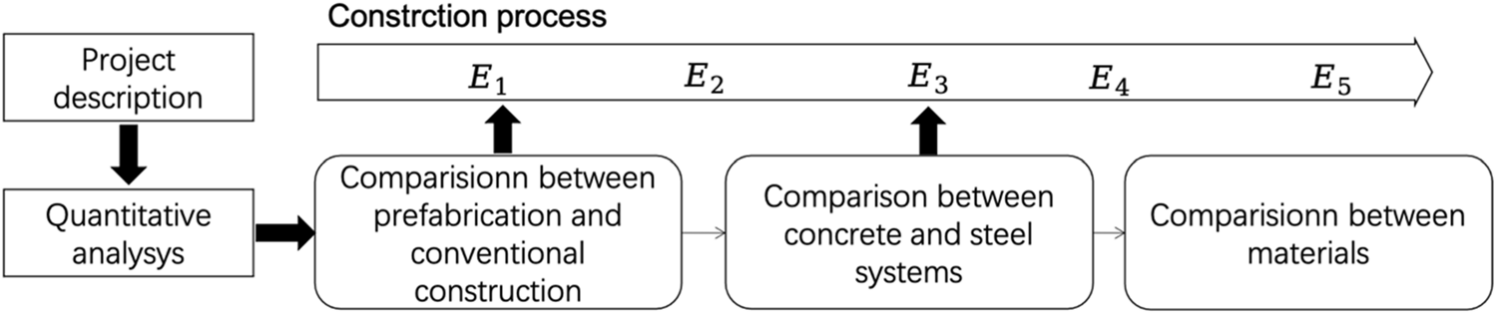
Graphical representation of the research route.

In this section, we conduct a case study of residential projects in Beijing, in order to validate the proposed evaluation method and examine the amount of GHG emissions between different construction methods and systems. We first describe the four projects under study, then conduct quantitative analysis in three dimensions: prefabrication and conventional construction, concrete and steel systems, and different materials (as shown in Fig. 2).

### Project description

**Figure 3:**
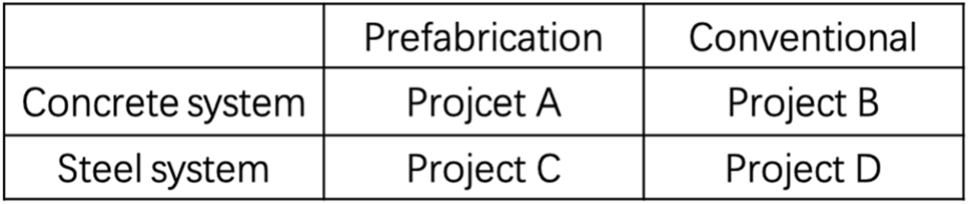
The classification of projects.

We present two groups of cases: the projects (Project A and Project B) in the first group are in a concrete structure, while the projects (Project C and Project D) in the second group are in steel structure (as shown in Fig. 3.). In each group, one project is built with a prefabricated construction method and the other one is built using the conventional method. Each group of projects is examined to evaluate the GHG emission and compared it to discover the differences between prefabrication and conventional methods. Furthermore, we also conduct a comparison between two structure systems: concrete and steel (the most popular structures in China), to find if there are apparent differences existing between the different materials.

**Figure 4:**
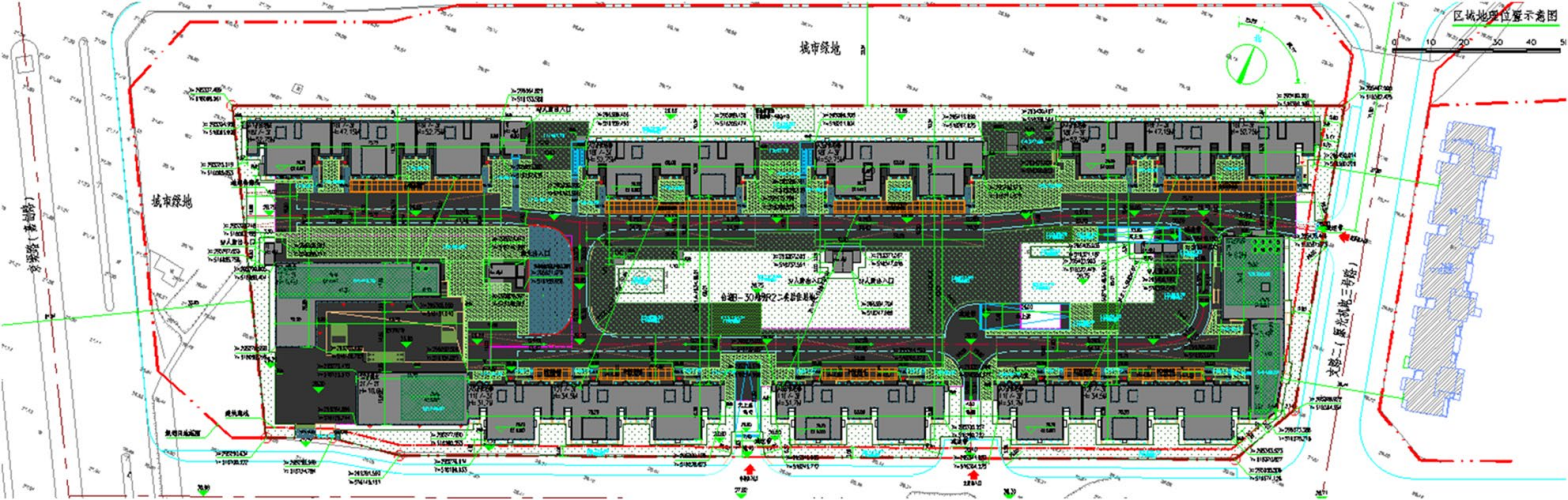
The general layout of project A.

**Figure 5:**
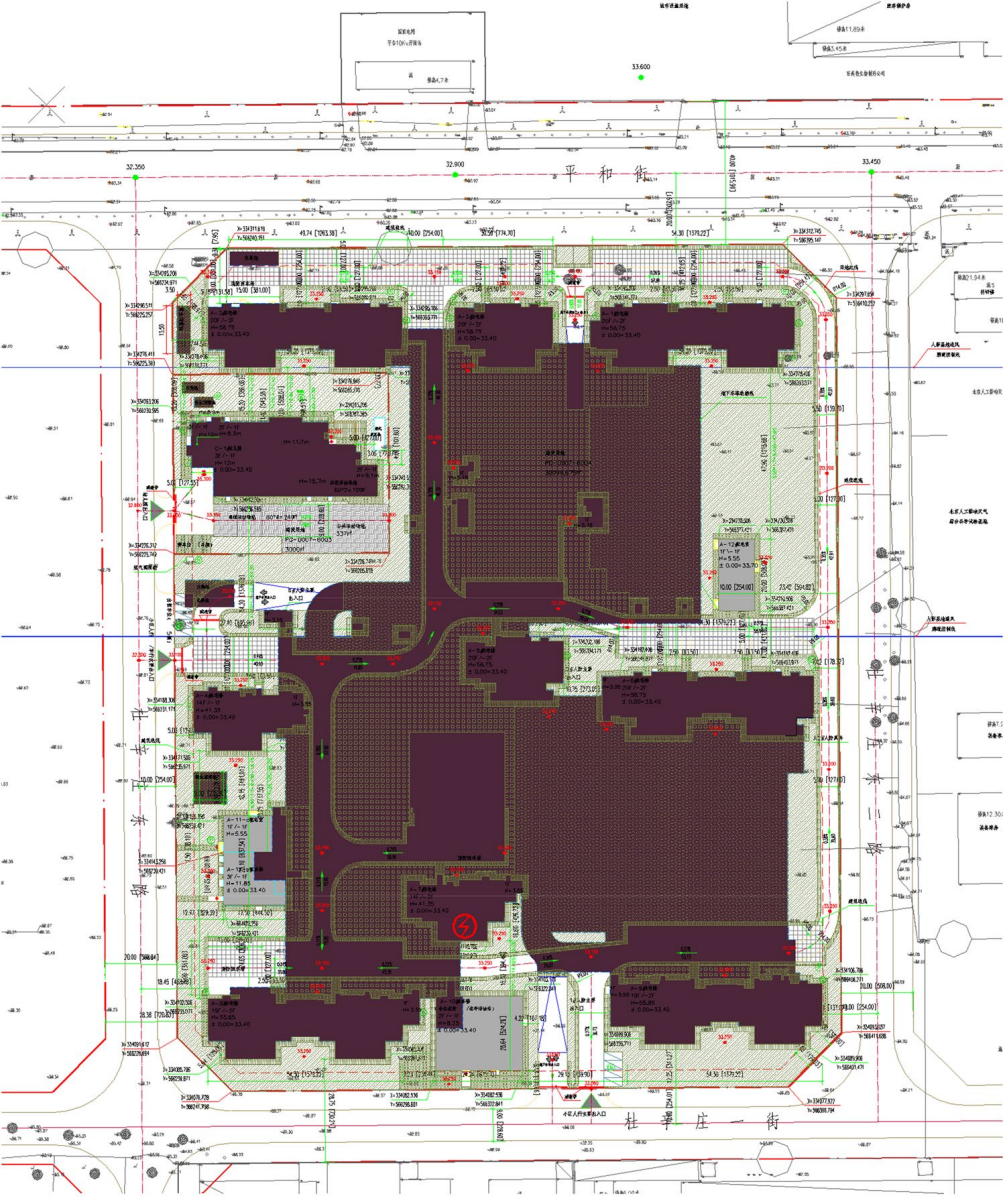
The general layout of project B.

**Figure 6:**
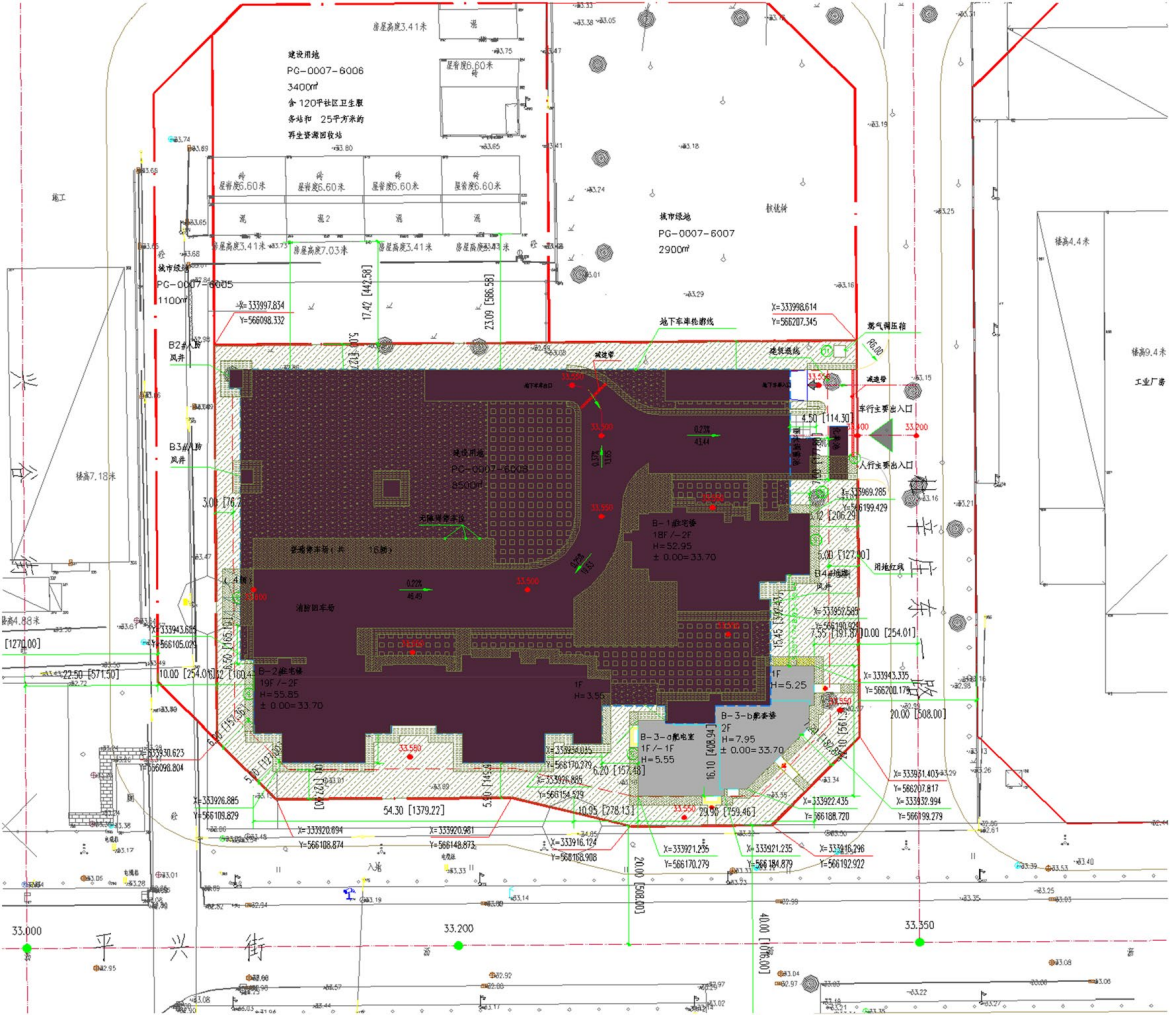
The general layout of project C.

**Figure 7:**
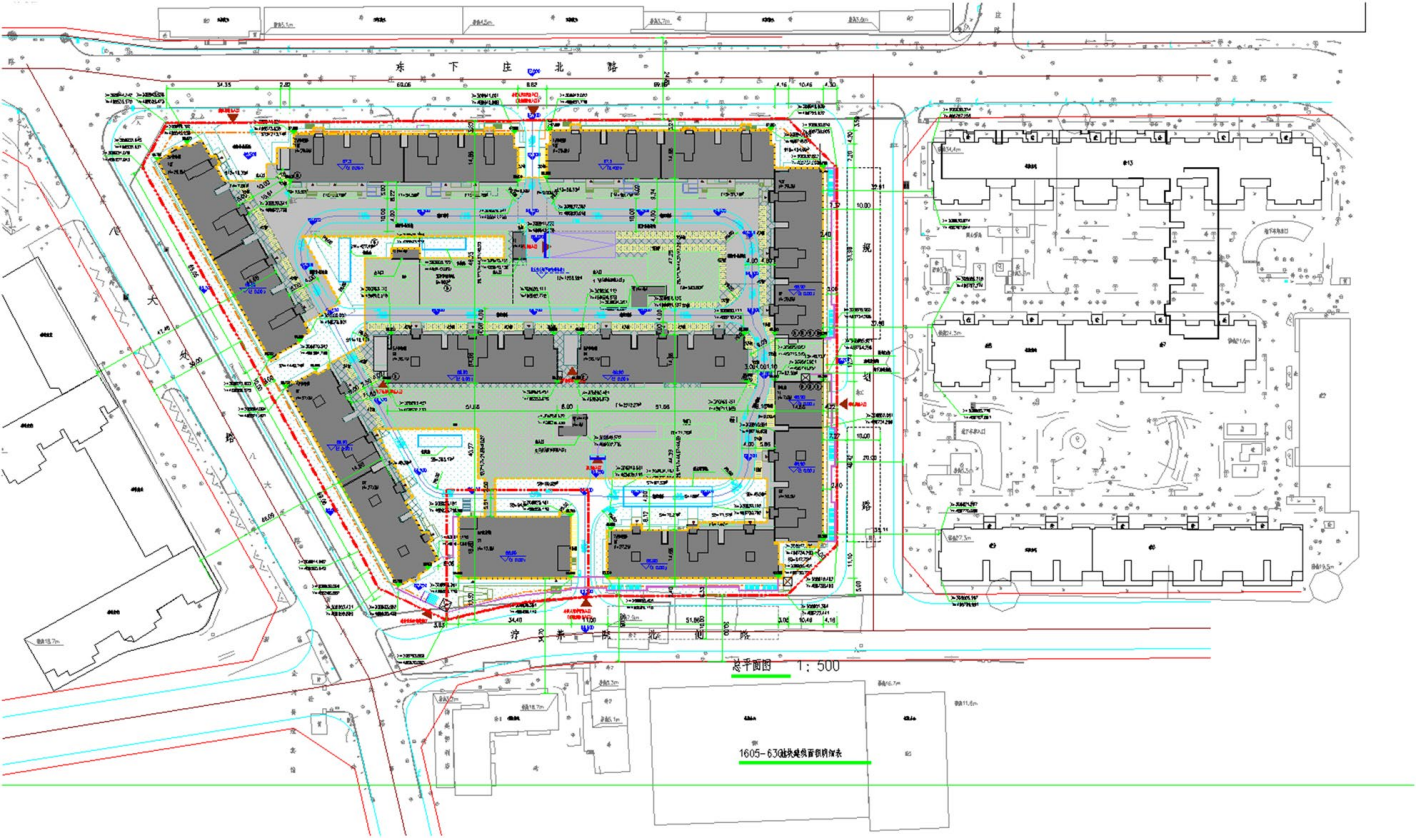
The general layout of project D.

Project A is a residential project in the Tongzhou district, Beijing. This project has the concrete structure building by semi-prefabricated construction method, where the first and second floors of the project is in cast-in-place concrete shear wall structures, and the third and higher floors have the components of prefabricated shear walls. The general layout of project A is shown in Fig. 4. Project B is another concrete structure residential project located in Tongzhou district, Beijing, adopting the conventional construction method (see Fig. 5). For the steel structure buildings, Project C is a kindergarten construction project in Pinggu District, Beijing, using prefabricated steel components, and project D is a steel-structure residential construction project in Pinggu District, Beijing, building by the conventional construction method, as shown in Figs. 6 and 7 respectively. A detailed description of this project is available in Table 4.

**Table 4:** The description of projects.

Items	Project A	Project B	Project C	Project D
Types	Residential, retail	Residential	Kindergarden	Residential
Location	Tongzhou	Tongzhou	Pinggu	Pinggu
Structure	Concrete	Concrete	Steel frame	Steel frame
Storeys	F19	F12	F4	F2
CFA	32,878 m^2^	28,591 m^2^	5204 m^2^	3783 m^2^

The data are collected through on-site surveys, project reviews, and interviews with project managers, prefabricated factories and material suppliers. In order to enhance the comparability of different projects, the scale of project B and D is adjusted with regard to project A and C, and renamed as project and respectively. The building materials of these project are focused on: ready-mixed concrete, cement, sand, steel, brick, glass, and the usage amount of each project is shown in Table 5.

**Table 5:** Building materials usage.

Building matrials	Using amount of Project A (ton)		Using amount of Project *B*′ (ton)	Using amount of Project C (ton)		Using amount of Project *D*′ (ton)
	Off-site	On-site		Off-site	On-site	
Ready-mix concrete	4259	36,399	39,059	1145	9159	9835
Cement	0	1989	2241	0	462	621
Sand	0	11,747	13,298	0	3095	4859
Steel	277	1732	1874	312	1951	2154
Brick	0	8021	9441	0	6417	2614
Glass	0	67	67	0	12	12

In this study, we suppose that these projects have the similar transportation distance (as shown in Table 6). Note that L^1^ is the distance from the off-site plant to the distribution center, and L^2^ is the distance from the construction site to the distribution center.

**Table 6:** The transportation distances of building materials.

Building materials	The distance from suppliers to prefab. (km)	The distance from suppliers to site (km)
Ready-mix concrete	5	80
Cement	–	60
Sand	–	60
Steel	50	120
Brick	–	15
Glass	–	10

**Figure 8:**
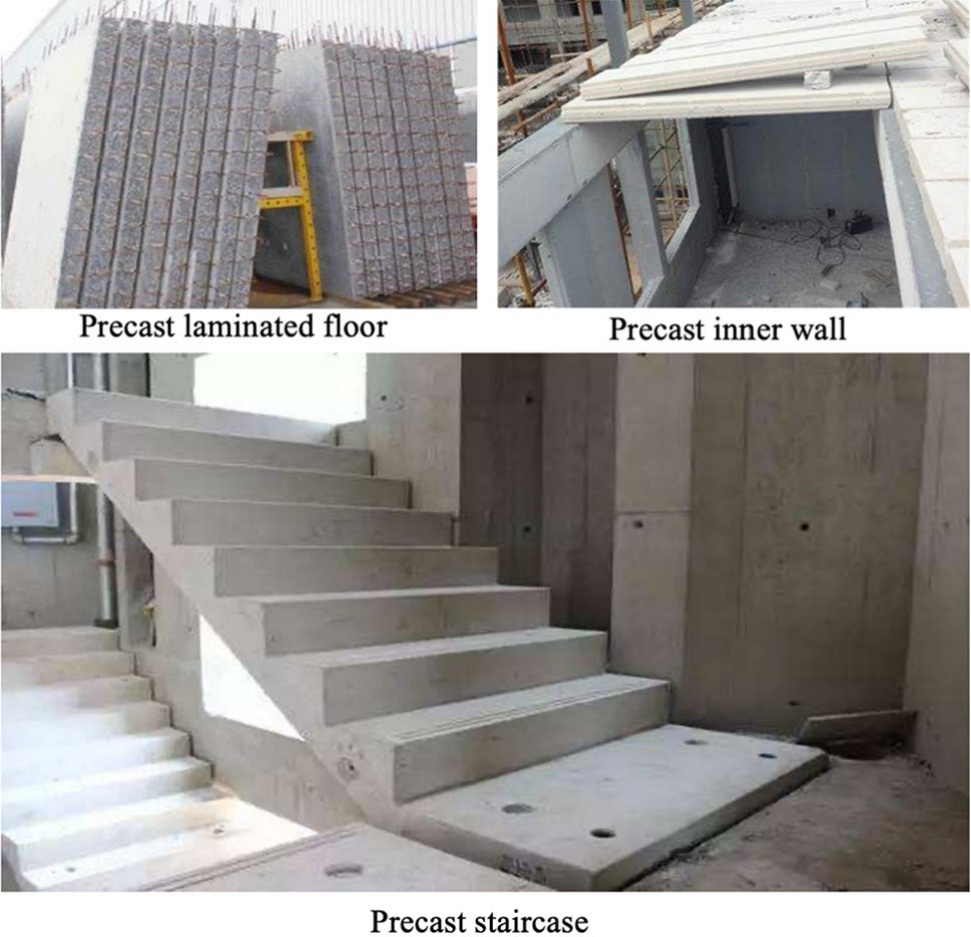
The three different types of prefabricated components of Project A.

**Figure 9:**
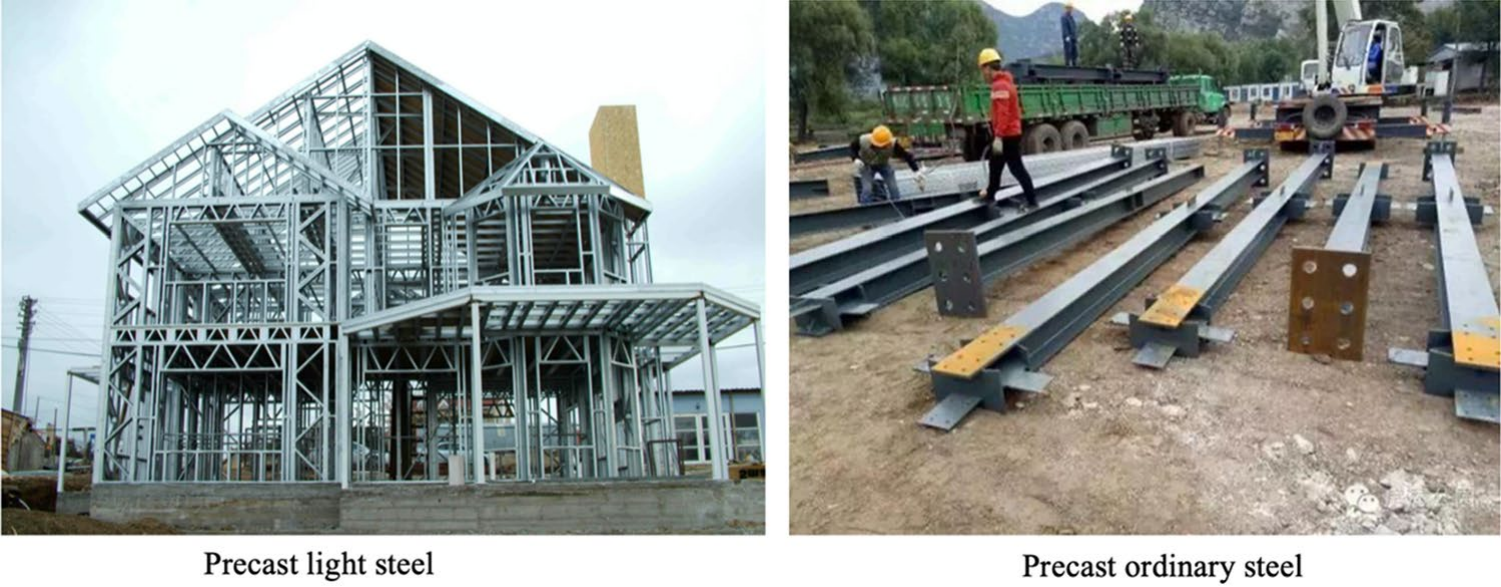
The two types of prefabricated components of Project C.

Project A has six types of prefabricated components (the three main components are shown in Fig. 8), and the prefabrication rate of the concrete structure exceeds 50%, and the total amount of prefabrication materials is 21,334 tons. Project C has a prefabrication rate of over 40% and has 5245 tons of prefabrication materials (the main components are shown in Fig. 9). The average distance between the prefabrication plant and the construction site (${L}^{1}$ for both Project A and Project C is assumed to be the same (which is 70 km). In this project, all of the materials, prefabrication, and construction waste are delivered by truck with a GHG emission coefficient of 0.207 (kg CO_2−e_/ton km). Table 7 summarizes diesel, electricity and water usage.

**Table 7:** The resource usage of construction activities on site.

Resource	The resource usage of Project A (unit)		The resource usage of Project *B*′ (unit)	The resource usage of Project C (unit)		The resource usage of Project *D*′ (unit)
	Off-site	On-site		Off-site	On-site	
Diesel (L)	1774	33,432	35,691	279	5430	5649
Electric (kWh)	16,282	325,632	376,105	2644	53,241	59,542
Water (m^3^)	4525	45,410	54,249	716	7187	8587

The distance from the site to the land-fill (${L}^{2}$) is 21 km, and the construction waste would be calculated by the waste rate of each building materials shown in the second column of Table 2.

### Results analysis

In this section, a quantitative analysis between concrete and steel structure system of both prefabricated and conventional construction methods is conducted. The corresponding total GHG emissions from different materials are compared to illustrate the effectiveness of the proposed evaluation method, and to fathom the environmental benefits of off-site prefabrication.

#### Analysis of total GHG emissions of prefabrication and conventional construction

The five major GHG emissions are calculated using Eqs. (1–5). The observations are as follows. For the concrete structure buildings, project A using a semi-prefabrication method has the total GHG emissions of 11,267.8 tons CO_2−e_, while project B with the conventional construction method has a total amount of 11,982.2 tons CO_2−e_. This renders a reduction of 714.4 tons CO_2−e_ (5.96% less than conventional method). The ninth column of Table 8 shows the proportional contribution in GHG emissions reduction by using the pre-fabrication method among the construction process. The most significant reduction (106.02%) of GHG emissions is contributed by the energy and resource saved on construction site (${\mathrm{E}}_{4}$). Besides, the second significant reduction occurs from the construction materials (${\mathrm{E}}_{1}$), which accounts for 41.2%. These two sources of GHG emission reductions renders the essential stages to reduce the environmental impact of residential projects by prefabrication. The fuel consumption occurs in the transportation stage (${\mathrm{E}}_{2}$) contributes a reduction of 13.1%. However, 60.2%, and 0.3% increase of GHG emissions are observed from the transport of prefabricated components (${E}_{3}$) and construction waste (${E}_{5}$) respectively. That is because prefabricated components’ transporting and the waste from the prefabrication factory are only calculated in prefabricated project and have a negative impact on GHG emissions reduction.

**Table 8:** GHG emissions in Project A (semi-prefabrication) and Project B (conventional).

Sources	Project A				Project *B*′		Reduction of GHG emissions		Percentage of GHG emissions reduction
			Total						
	Off-site	On-site	(S)	%	(T)	%	(T-S)	(CP) %	[Z = (T−S)/T] %
El	612.8	8571.1	9183.9	81.50	9478.2	79.10	294.3	41.20	3.10
E2	10.1	1170.7	1180.8	10.50	1274.2	10.60	93.4	13.10	7.30
E3	430.1	0	430.1	3.80	0	0	− 430.1	− 60.20	0
E4	23.1	437.8	460.9	4.10	1219.6	10.20	758.7	106.20	62.20
E5	2.8	9.3	12.1	0.10	10.2	0.10	− 1.9	− 0.30	− 18.60
TGE			11,267.8		11,982		714.4		

Similar results for the steel structure buildings are observed (as shown in the Table 9). Project C and D have the total GHG emissions of 4677.9 tons CO_2−e_, and 5464.3 tons CO_2−e_, respectively. A reduction of 786.4 tons is acquired by using the semi-prefabrication method. The biggest part of emissions reduction is from the energy of materials (${E}_{1}$), the construction materials’ transporting (${E}_{2}$) is the second large part of emissions reduction, which is different from the concrete structure buildings.

**Table 9:** GHG emissions in Project C (semi-prefabrication) and Project D (conventional).

Sources	Project C				Project *D*′		Reduction of GHG emissions		Percentage of GHG emissions reduction
			Total						
	Off-site	On-site	(S)	%	(T)	%	(T−S)	(CP) %	[Z = (T−S)/T] %
El	251.9	3760.1	4012	85.80	4655.6	125.60	643.6	90.10	13.80
E2	6.1	367.7	373.8	8.00	610.6	16.50	236.8	33.10	38.80
E3	105.7	0	105.7	2.30	0	0.00	− 105.7	− 14.80	0.00
E4	9.5	172.4	181.9	3.90	193.1	5.20	11.2	1.60	5.80
E5	1	3.5	4.5	0.10	5	0.10	0.5	0.10	10.00
TGE			4677.9		5464.3		786.4		

#### Analysis of GHG emission of concrete and steel systems

For projects with prefabrication, as we can observe from Tables 8 and 9, for both concrete and steel systems, the largest amount of GHG emissions occur in the stage ${\mathrm{E}}_{1}$ (construction material), 81.5% and 85.8%, respectively. The other sources of GHG emissions have similar percentages.

For project with conventional construction, the largest GHG emissions occur in stage ${\mathrm{E}}_{1}$ as well. The second largest source of GHG emission is three times than the third largest source, i.e., the value is 16% (${\mathrm{E}}_{2}$) and 5.2% (${\mathrm{E}}_{4}$) for steel structure. However, for concrete system, the GHG emissions difference between stage ${\mathrm{E}}_{2}$ and ${\mathrm{E}}_{4}$ is not significant. This illustrates that for concrete system, the on-site construction is an energy intensive stage compared with steel systems.

Overall, the source of ${E}_{4}$ for concrete structure significantly benefits from semi-prefabrication in comparison to other sources, as shown in the tenth column of Table 8. By contrast, the largest reduction of GHG emissions for the steel structure is from the sources of ${E}_{5}$ and ${E}_{1}$ as indicated in the tenth column of Table 9.

#### Analysis of GHG emission from different construction resources

For concrete structure buildings, namely, project A and B, the adopt of semi-prefabrication has an average reduction of 294.35 tons CO_2−e_ GHG emissions. Both project A and project B have the largest usage amount of concrete and have the contribution of 53.12% and 49.45% for the GHG emissions, respectively. The disparity of the construction materials usage amount between conventional construction methods and the semi-prefabrication is significant. That is because the techniques, design requirements and construct processes of the prefabrication method are different from the conventional construction method^6^. For both construction methods, the GHG emissions (${E}_{1}$) of each material change according to the using amount. It can be seen from Table 9 that the use of prefabrication methods reduces the consumption of materials such as concrete, steel, and glass (with little change). This is mainly because of the design requirements, especially for on-site manufacturing. Many connecting parts of steel bars are embedded in prefabricated walls, stairs, and floor slabs. The analysis of embedded parts shows that during the prefabrication process, GHG emissions from steel have increased. The increase of GHG emissions from concrete is due to the use of prefabricating concrete instead of traditional bricks in the external walls. Considering project C and D, a reduction in GHG emissions of 643.68 in CO_2−e_ is achieved because of the prefabricated construction method. Specifically, the contribution rates for materials in project C are 39.35% for brick, 30.82% for concrete, 20.70% for steel, 8.04% for cement, 0.54% for glass, and 0.54% for sand. The respective percentages for project D are 20.72%, 38.03%, 25.47%, 13.97%, 1.1%, and 0.72%.

There are three types of greenhouse gas emissions related to transportation activities, namely, ${E}_{2}$, ${E}_{3}$ and ${E}_{5}$. For concrete structures, transportation accounts for 14.4%, while traditional methods account for 10.7%. This is because the transportation of prefabricated components and waste from prefabricated factories only exists in off-site prefabrication. As long as ${E}_{4}$ is reduced, the total greenhouse gas emission capacity can be raised to a higher level. One potential way to reduce greenhouse gas emissions in unnecessary transportation is, therefore, only by minimizing transportation distances to reduce greenhouse gas emissions.

Comparing these two groups of buildings (concrete structure and steel frame structure), the results show that traditional construction methods produce more greenhouse gases than prefabricated construction methods. For concrete structure buildings, the amount of greenhouse gases is reduced by 62.2%, while for steel frame structures, the amount of greenhouse gases is reduced by only 12%. For concrete structure buildings, 1.4% diesel, 64% electricity, and 85% water have been reduced respectively. For the steel structure buildings, except for a little increase in the usage of diesel, the rest are reduced, for  − 1%, 6%, and 8%, respectively. The reasons are the same as mentioned above. The significant reduction of resources using amount is due to the standardized off-site factory-built process, and the effective combination of construction links and interfaces, thereby reducing unnecessary waste.

## Conclusions

The construction process of residential projects generates a large quantity of GHG emissions. Different building methods emits different amount of GHG emissions. As a frequently used construction method, prefabrication attracts more and more research attentions. In this study, we focus on prefabrication method with concrete and steel systems. We propose a process based approach to evaluate the environmental impact of residential projects with different systems. In order to characterize and quantify the GHG emissions, the system boundaries for the evaluation are defined and then a quantitative model of GHG emissions based on the entire construction process is given. The total GHG emissions of four residential buildings with different structures and construction methods are compared and managerial insights are given.

The comparative analysis among the projects shows that different construction methods produce different amounts of GHG emissions. Results indicate that the project with prefabrication reduced around 714.4 tons CO_2−e_ for concrete systems and 786.4 tons CO_2−e_ for steel systems. For both concrete structure buildings or steel frame structure buildings, adopting the prefabricated construction method can significantly reduce GHG emissions. Furthermore, the choices of main building resources have a great impact on GHG emissions as well. The usage of prefabricated components in concrete structure buildings could reduce GHG emissions significantly, however, there is less reduction for the steel structure buildings by using the prefabricated methods.

In this study, we propose a quantitative approach to evaluate the amount of GHG emissions in prefabrication projects with concrete and steel systems. We also illustrate the proposed approach on four residential projects recently completed. Our case analysis reveals that prefabrication and conventional construction methods with different systems contribute differently to emissions. Prefabrication generates less emission compared to conventional construction methods. The reduction is 6.4% for concrete system and 16.8% for steel system. The proposed approach can be applied to other residential projects with different construction methods and structures. The managerial insights revealed from the case study are instructive for relevant stakeholders, i.e., policy makers, contractors and residents in the future. Finally, it would be interesting to conduct multiple evaluation of the correspondence between construction methods and structure systems.

## Data Availability

The datasets generated and/or analyzed during the current study are available from the corresponding author on reasonable request.
