# Author Correction: System based greenhouse emission analysis of off-site prefabrication: a comparative study of residential projects

**DOI:** 10.1038/s41598-024-56778-9

**Published:** 2024-03-15

**Authors:** Yuliang Guo, Enhui Shi, Rui Yan, Wenchao Wei

**Affiliations:** 1 School of Economics and Management, Beijing Jiaotong University, Beijing, China; 2 School of Economics and Management, University of Science and Technology Beijing, Beijing, China

Correction to:* Scientifc Reports*, 10.1038/s41598-023-37782-x, published online 1 July 2023

The Funding section in the original version of this Article was omitted. The Funding section now reads:

“Fundamental Research Funds for the Central Universities with Grant Number 2020JBW004.”

The original Article has been corrected.

